# Validation of the CIMI-Ethiopia Program and Seasonal Variation in Maternal Nutrient Intake in Enset (False Banana) Growing Areas of Southern Ethiopia

**DOI:** 10.3390/ijerph16162852

**Published:** 2019-08-09

**Authors:** Tafese Bosha, Christine Lambert, Simon Riedel, Ute Gola, Aberra Melesse, Hans K. Biesalski

**Affiliations:** 1 Institute of Nutritional Sciences, University of Hohenheim, Garbenstr. 30, 70593 Stuttgart, Germany; 2 College of Agriculture, Hawassa University, P.O. Box 5, Hawassa, Ethiopia; 3 Day Med Concept GmbH, Garbatyplatz 2, 13187 Berlin, Germany

**Keywords:** CIMI-Ethiopia, lean wet season, micronutrient intake, postharvest dry season, reproductive age

## Abstract

Background: Tools for the rapid and accurate analysis of nutrient intakes from diets of individuals in Southern Ethiopia are lacking. The Calculator of Inadequate Micronutrient Intake program for Ethiopia (CIMI-Ethiopia) has been developed to overcome this problem. CIMI-Ethiopia also computes protein and energy intakes from the diet. The objectives of the current study were to validate CIMI-Ethiopia for the dietary pattern of Southern Ethiopia, and assess the nutrient intakes in postharvest dry and lean wet seasons. Methods: 24-h dietary recall (24HR) data was collected from 578 women of a reproductive age in postharvest dry and lean wet seasons in 2017. For analysis, 24HR data was entered into NutriSurvey (NS), which was the reference nutrition software, and then into CIMI-Ethiopia. For validation, the mean and standard deviation (SD) of the difference between CIMI-Ethiopia and NS were computed. The percentage of participants with an inadequate intake was calculated. The correlation between CIMI-Ethiopia and NS results was determined. The nutrient intakes in postharvest dry and lean seasons were compared. Results: Among the nutrients, pantothenic acid, vitamin B1, and protein showed a very high accuracy in CIMI-Ethiopia calculation (|difference (D)| < 5.0% of the NS result). Nutrients with a good accuracy (|D| = 5%–15%) were iron, zinc, magnesium, vitamin B12, vitamin B6, and energy. The accuracy for calcium, niacin, and vitamin A was moderate (|D| = 15%–30%). The intakes calculated by CIMI-Ethiopia and NS of iron, zinc, magnesium, calcium, B-complex vitamins, vitamin A, protein, and energy were highly correlated (r = 0.85–0.97, *p* < 0.001). NS analysis identified a significant reduction in the mean intake of iron; zinc; magnesium; pantothenic acid; vitamin B1, B12, and D; protein; and energy in the lean wet season; however, calcium and vitamin A intake increased. Conclusions: It has been found that CIMI-Ethiopia is a valid tool for estimating nutrient intakes at an individual level in Southern Ethiopia. The study demonstrated a decline in intakes of iron; zinc; magnesium; pantothenic acid; vitamin B1, B12, and D; protein; and energy in the lean wet season. This result provides some hint for fortification and supplementation programs that aim to combat maternal malnutrition in rural Southern Ethiopia.

## 1. Introduction

Micronutrient deficiencies (MND) are of high public health and socioeconomic importance worldwide [1]. Women of a reproductive age, specifically pregnant and lactating mothers, are among the population segment that is most vulnerable to MND [2]. MND, also called hidden hunger, is caused by a poor dietary nutrient intake, disease, or increased micronutrient needs not met during pregnancy and lactation [3]. In the present study zone, where *enset* (false banana [*Ensete ventricosum*]) is a staple food and the main crop [4], deficiencies of micronutrients such as zinc [5] and vitamin A [6] have public health significance. Besides, limited small-scale studies reported a high prevalence of an inadequate intake of zinc and calcium in pregnant women from the same agro-ecology [7]. Moreover, a similar prior study reported very low protein and energy intakes [7]. However, there is a lack of information on the seasonal trends of nutrient intake.

*Enset* provides staple food for ~20 million Ethiopians [8]. *Kocho, bulla,* and *amicho* (corm) are the major food products of *enset* [9]. Of the three, the main product is *kocho*, a fermented bread-like food that is consumed locally, as well as sold to urban markets [4]. It is a mixture of scraped pulp of the pseudo stem and pulverized corm of *enset* [10]. *Bulla* is a high-grade product prepared by squeezing and dehydrating the juice from parenchymatic scrapings of the pseudo stem, which is tightly packed in *enset* leaves and fermented in a silo [11]. However, food products from *enset* are relatively nutrient poor. For example, *bulla* contains 7.58 mg iron, 0.22 mg zinc, and 0.65 g protein, while *amicho* contains 13.04 mg iron, 23.65 mg zinc, and 8.8 g protein, on a dry weight basis. On the contrary, teff (*Eragrostis teff*) provides 22.37 mg iron, 4.74 mg zinc, and 14.58 g protein on a dry weight basis per 100 g [9]. Additionally, *kocho* is poor in protein. For instance, a *kocho*-based complementary food provides only 1.46 g protein per 100 g on a dry weight basis [12].

While assessing the nutrient quality of the diet is essential, and the first dimension of preventing deficiencies, there is a lack of a rapid tool for performing a timely assessment. Existing methods, for instance, dietary history, are expensive and time consuming, and a food frequency questionnaire (FFQ) is specific to study groups and research aims, and suffers from measurement errors related to methodology [13]. 

The Calculator of Inadequate Micronutrient Intake (CIMI) program has been found to be a rapid solution to assess individuals’ nutrient intake. The reason for this timely result of CIMI is that the dietary consumption data entry and analysis are automated in this device. Moreover, CIMI considers the bioavailability of iron and zinc in individual diets. Apart from micronutrients, CIMI computes the protein and energy intake [14,15,16,17,18]. 

CIMI was firstly developed for the Indonesian dietary pattern [14], and then Ghanaian [15,16] and Tanzanian [17]. Recently, it was adapted to Ethiopia and CIMI-Ethiopia was released as a mobile application for use with a tablet, which has been validated in the Tigray Region of Northern Ethiopia [18]. However, the validation of CIMI-Ethiopia for the dietary pattern of Southern Ethiopia needs further investigation. This is mainly because of the variation in the dietary pattern in the two regions. More specifically, the low dietary diversity in the Tigray Region, which is 2.5 in fasting to 3.1 in non-fasting seasons [19], may not sufficiently address all food groups (31) in the current version of CIMI-Ethiopia. Besides, diets in Southern Ethiopia contain more roots and tubers, but less cereals and grains, compared with the Tigray Region [20]. 

Previous studies compared CIMI against NutriSurvey (NS), which is an established nutrition analysis software [14,15,16,17,18]. The current study also used NS as a reference software to compare the nutrient results computed by CIMI-Ethiopia. This is because NS represents the method most commonly used to analyze data from dietary assessment methods such as 24-h dietary recall (24HR) [14]. NS is the English translation of the professional German nutrition software EBISpro, which contains functions for nutrient analysis and the calculation of energy requirements [21]. NS assesses individual foods with regard to the portion size in grams in the diet and their respective nutrient content. On the other hand, CIMI-Ethiopia considers specific food groups with regard to the portion size in the diet. It computes nutrient intakes and the percentage of nutrient fulfillment in comparison with the FAO/WHO recommended nutrient intake (RNI) [22,23,24]. Therefore, the present study was designed to validate CIMI-Ethiopia for the diets of Southern Ethiopia and assess the maternal nutrient intakes in post-harvest dry and lean wet seasons.

## 2. Materials and Methods 

### 2.1. Study Area

This study was conducted in Shebedino and Hula Districts from Sidama Zone in the Southern Nations Nationalities and Peoples Region (SNNPR). The regional capital city Hawassa is located nearly 278 km south of the national capital Addis Ababa. Sidama Zone has a total population of nearly 3.1 million [25]. *Enset*, maize, and coffee are among the major crops in Sidama Zone. *Enset* is a crucial drought-resistant crop with significant cultural value in the zone [26]. Shebedino and Hula are two of nineteen districts and two city administrations of the zone [27]. Nearly 99% of the households in the five kebeles (kebele is the smallest administrative unit of a district) sampled from the two districts produced *enset* in the l2 months before the survey [28]. 

### 2.2. Study Design

This is a longitudinal study in which food and nutrient intake data were collected in postharvest dry (January to first week of February 2017) and lean wet seasons (June 2017). 

### 2.3. Study Population 

All women of a reproductive age with 24–59-month-old children were eligible for this study. In order to reduce those lost to follow-up, temporarily resident mothers were not included in the study. Besides, women were excluded from the survey when reported as being severely sick to avoid the effect of illness on the dietary intake due to a reduced appetite.

### 2.4. Sample Size Calculation 

For this particular analysis, a basic sample size of 319 was computed using a correlation coefficient (r) with the following formula [29], assuming a two-tailed test: *α* (type I error rate), 0.050; *β* (type II error rate), 0.050; and r (expected correlation coefficient), 0.20.

$$N={\left[\frac{Z\alpha +Z\beta }{C}\right]}^{2}+3$$

Here, *N* = sample size; *α* = *Z_α_* = 1.960; *β* = *Z_β_* = 1.645; and *C* = 0.5 * ln[(1 + r)/(1 − r)] = 0.203. On top of this, the design effect of 1.5 was considered, and 10% of the basic sample size was added to compensate for those lost to follow-up, resulting in a total of 510. However, a total of 625 mothers with 24–59-month-old children were enrolled at the beginning in order to obtain a sufficient sample size for additional analysis [28]. Nonetheless, 578 completed the study, while the rest left for different reasons: death (1), delivery (4), and sickness or moving to other places (42). 

### 2.5. Sampling 

A two-stage sampling technique was employed. Firstly, Shebedino and Hula Districts were selected from the Sidama Zone. Secondly, Fura, Howolso, Dila Gumbe, Worare, and Chirone Kebeles were randomly selected using probability proportional to size (PPS) from the two districts. Then, the sample size was distributed to each kebele using PPS. The samples were randomly selected based on the sampling frame prepared from a house-to-house listing of households with 24–59-month-old children [28]. 

### 2.6. Data Collection

Ethical clearance was received from the Institutional Review Board of Hawassa University, Ethiopia, and Ethik-Kommission, Landesäztekammer Baden-Württemberg, Germany (F-2016-127). Permission was obtained from health administrative offices of the study districts to access the study population and collect data. Informed written consent was received from mothers. Information was kept confidential with pseudonymous codes.

Data collectors with previous experience were recruited. The principal investigator trained the data collection staff for one week. The same data collection team collected the lean season data after additional project-specific training. The pre-test of survey tools was carried out on five percent of the total sample size to check for appropriateness. Data collection was supervised by the principal investigator.

A structured questionnaire was first prepared in English, and then translated to Amharic. The purpose of this questionnaire was to collect the sociodemographic data. A standard 24HR protocol was used to collect the dietary intake in the 24 h preceding the survey. To assess the usual dietary nutrient intake, the dietary consumption data was collected on days other than fasting and holidays. In order to facilitate mothers’ recall, food models and food charts were used. The amounts of foods and beverages consumed were estimated in terms of local units: coffee cup, ladle, soup spoon, water glass, and tea spoon. Printed pictures aided to identify portion sizes, for instance large or medium or small, for countable food items like potato. Data collectors probed the mothers to remember all food items consumed in the 24 h preceding the survey. A compact scale CS2000 (Ohaus Corporation, Parsippany, NJ, USA) was used to convert the weight equivalents of the local units into grams of foods consumed.

### 2.7. Statistical Analysis

Before data entry, the uniformity of the nutrient compositions of foods in CIMI-Ethiopia and NS were assured by adopting the compositions configured in the prior one. Following this, the dietary consumption data collected with 24HR was entered into NS 2007, which is a reference program used to compute nutrient intakes. Then, nutrient data was exported from NS into Microsoft Excel 2016, and subsequently into Statistical Package for Social Sciences (SPSS) ver. 20 (IBM Corporation, Armonk, NY, USA) for analysis. Secondly, the same dietary intake data from 24HR was entered into CIMI-Ethiopia. The nutrient intake data calculated by CIMI-Ethiopia was automatically transferred to a server when WIFI was available. Later, the data was downloaded from the server and imported into SPSS. Thirdly, the sociodemographic data was entered directly into SPSS. Then, the data sets were compiled together and checked for completeness before analysis. One participant was excluded from this analysis, as the amount of the food consumed was missing, though types of foods were recorded. Data normality was checked with the Kolmogorov–Smirnov test. For validation, the mean and standard deviation (SD), and median with 25th and 75th values of intakes, were calculated for iron, zinc, calcium, magnesium, vitamin A, vitamin B1, niacin, pantothenic acid, vitamin B6, vitamin B12, vitamin D, protein, and energy from CIMI-Ethiopia and NS. The Pearson’s test was used to determine the correlation between the nutrient results from CIMI-Ethiopia and NS. Furthermore, a Bland–Altman plot was used to determine how well the data fit. Besides, the percentage of participants below the threshold of an inadequate intake was computed. The nutrient intake less than two-thirds of the FAO/WHO RNI [22,23,24] was taken as a cutoff point for the threshold of an inadequate intake [14,30,31]. To determine the level of accuracy, the mean and SD of the difference between the intake computed by CIMI-Ethiopia minus the intake calculated by NS were computed. Accordingly, an absolute difference, i.e., |D (difference)| < 5.0% of the NS result was categorized as a very high accuracy, |D| = 5%–15% as a good accuracy, |D| = 15%–30% as a moderate accuracy, and |D| > 30% as a low accuracy [17]. In order to determine the seasonal differences of nutrient intakes, the Wilcoxon signed-rank test was used to compare non-normally distributed mean nutrient intakes between postharvest dry and lean wet seasons computed by NS. The cutoff point for statistical significance was a *p*-value ≤ 0.05.

## 3. Results

### 3.1. Sociodemographic Characteristics of Participants

Table 1 shows the sociodemographic characteristics of study participant mothers. Nearly 89% of the mothers were 25–34 years old. Around 75% of them were lactating during the postharvest dry season; however, this percent was decreased to 59% in the lean wet season. Nearly 42% of the mothers were illiterate and 88% were housewives.

### 3.2. Validation of CIMI-Ethiopia 

Table 2 demonstrates the micronutrient intake in postharvest dry and lean wet seasons as computed by CIMI-Ethiopia and NS. It was found that the results computed by the two methods were closely comparable in terms of all the three parameters assessed, i.e., mean and SD, median with the 25th and 75th percentile, and number and percent of participants below the threshold of an inadequate intake for most of the nutrients. The differences seen in vitamin A and calcium intakes were also in an acceptable range. 

**Figure 1:**
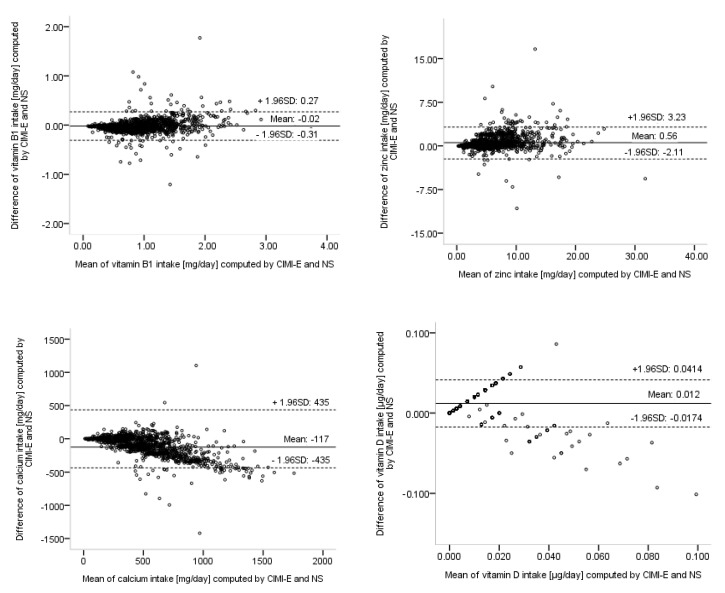
Bland–Altman plots for vitamin B1, zinc, calcium, and vitamin D representing nutrients with a very high, good, moderate, and low accuracy, respectively (N = 1154). Solid line represents mean bias; dotted lines represent ± 1.96SD (limit of agreement); CIMI-E: Calculator of inadequate micronutrient intake program for Ethiopia; NS: NutriSurvey.

Table 3 demonstrates the average mean and SD of the difference between the intake computed by CIMI-Ethiopia minus the intake calculated by NS in two seasons, and the percent difference. The table also shows the Pearson’s correlation between the nutrient intakes calculated by CIMI-Ethiopia and NS. The results indicate that the differences in nutrient intakes between CIMI-Ethiopia and NS were marginal. Among the nutrients investigated, pantothenic acid, vitamin B1, and protein had a very high accuracy (|D| < 5.0% of the NS result). Nutrients with a good accuracy (|D| = 5%–15%) were iron, zinc, magnesium, vitamin B12, vitamin B6, and energy. The accuracy for calcium, niacin, and vitamin A was moderate (|D| = 15%–30%). Correlations between nutrient intakes computed by the two devices were very strong, except for vitamin D (Table 3), which is further depicted by Figure 1. 

The Bland–Altman plots of vitamin B1 and zinc intake (Figure 1) show a dispersion of the calculations around the zero value of the y-axis, indicating a good fitness between the NS and CIMI analysis, independent of the intake amount. However, for calcium and vitamin D, underestimation by CIMI increases with higher intake amounts. The different dispersion pattern in the plot of vitamin D is due to the low number of participants reporting a consumption of vitamin D-containing foods/food groups.

### 3.3. Seasonal Variation in Nutrinet Intake 

From the nutrients studied, the mean intake was significantly reduced for iron; zinc; magnesium; pantothenic acid; vitamin B1, B12, and D; protein; and energy in the lean wet season. However, calcium and vitamin A intakes were significantly increased in the lean wet season. The intake of niacin and vitamin B6 did not change with season (Table 4). 

## 4. Discussion

### 4.1. Validation of CIMI-Ethiopia 

The present study investigated whether or not the CIMI-Ethiopia program validly assesses nutrient intakes from diets of Southern Ethiopia. An assessment of the seasonal variability in the maternal nutrient intake was also part of the study. The results are discussed below, accordingly.

A study from Indonesia reported a positive correlation between CIMI and NS in iron (r = 0.824), zinc (r = 0.608), vitamin A (0.686), protein (0.919), and energy (0.907) [14]. A further study from Ghana reported a higher correlation in iron (r^2^ = 0.966) [15]. The precision of CIMI-Ethiopia for the Southern Ethiopia diet is better than the report from Indonesia for all nutrients assessed, but comparable with that of Ghana for iron and other nutrients, except for vitamin A [15,16]. A possible explanation for the discrepancies in the precision may be associated with the variation in the number of food groups, i.e., 13 for Indonesia [14], 23 for Ghana [16], and 31 for CIMI-Ethiopia. The larger number of food groups in the present study may have helped in a more specific grouping of food items. More briefly, a careful food grouping is highly important for CIMI precision as it computes nutrient profiles group-wise. For instance, in the current version of CIMI for Ethiopia, red and white teff are in separate groups, even though the two items are varieties of the same grain. The reason for this is that red teff is iron-rich, but white teff has a relatively lower content. Therefore, had the two teff varieties been in one group, the precision for the iron result would have been reduced compared to the present record (Table 3). However, this is not a problem with its counterpart, i.e., NutriSurvey (NS), as it computes the nutrient intake for individual food items. 

Lambert and colleagues from Tanzania reported a very high accuracy for vitamin B6, protein, and energy; a good accuracy for vitamin B1, iron, and zinc; and a moderate accuracy for vitamin A and B12, and calcium [17]. In the current study, pantothenic acid, vitamin B1, and protein showed a very high accuracy; however, iron, zinc, vitamin B6, and energy displayed a good accuracy (Table 3). The variations between the two reports could be attributable to the difference in the dietary pattern of the two countries. Despite that, the accuracy for calcium and vitamin A was recorded to be moderate (Table 3), which is concurrent with the earlier report [17]. A parallel research study conducted on fasting and non-fasting Christians in the Tigray Region reported a very high accuracy for iron, zinc, calcium, magnesium, vitamin B1 and B12, pantothenic acid, and vitamin D [18]. Additionally, a similar study found a good accuracy for niacin, and vitamin A and B6 [18]. The results from the Tigray and Southern Regions are highly consistent for pantothenic acid, protein, and vitamin B1 and B6, and comparable for others. The first explanation for the disagreements is a variation in the dietary pattern between the two regions, while the second is the data entry mode in CIMI-Ethiopia. More precisely, diets of the Southern Region contain many more roots and tubers compared with that of Tigray [20]. In addition, recently published research on the same population showed that the consumption prevalence of dark green leafy vegetables was 53.6% in the postharvest dry season and 80.3% in the lean wet season [28]. This figure is considerably higher than the report from Tigray in fasting and non-fasting periods, where only 11% of the participants ate them [19]. Therefore, a more frequent presence of such food items in the daily diets of the current study participants has raised their total amount consumed in the preceding 24 h. However, the data entry mode of CIMI-Ethiopia is not flexible for the entry of higher amounts reported, especially after the first eight levels in an increasing order of the quantities eaten. The current version of CIMI-Ethiopia has 15 nonlinear stop point sliders for data entry. Therefore, after the first eight levels of quantities, the level of sliders for entry points increases by 1.0 quantity from 8.0 to 15.0 for most of the food groups. Therefore, this requires the use of mathematical rounding off to the nearest level in the case of higher quantities of foods groups consumed. This systematic error could be responsible for a negative deviation in the accuracy of nutrient results when compared with the standard program NS. This implies that a further improvement in the data entry mode of CIMI-Ethiopia could enhance the accuracy for diets of Southern Ethiopia, and enhance it for other regions too. Additionally, the dietary diversity is relatively higher in Southern Ethiopia, with the mean dietary diversity score (DDS) ranging from 3.20 and 3.26 in the postharvest dry and lean wet season [28], compared with the Tigray Region. For instance, a recent study from Tigray showed that grains are major staples in the area, with DDS varying from 2.57 to 3.0 in fasting and non-fasting periods [19]. The higher the dietary diversity, the more the number of food groups assessed in CIMI. This, in turn, may result in a possible reduction in the CIMI’s accuracy, as it computes the nutrient profile for food groups, unlike that of its counterpart program NS, which calculates values for individual food items. Based on this hypothesis, we recommend further validation studies in other regions of Ethiopia on various cultural dietary patterns.

Vitamin D intake is extremely low in diets of the study participants. Therefore, the variations between CIMI-Ethiopia and NS could not be large enough to affect percent intakes, i.e., number of participants below the threshold of an inadequate intake (Table 2) rather than the absolute intake, resulting in a lower correlation (Table 3). However, CIMI-Ethiopia tends to underestimate when the intake increases (Figure 1). Improvement in the data entry mode of CIMI-Ethiopia could improve the accuracy of the vitamin D calculation in the study population.

Overall, the closely comparable mean and median intakes, percentage of participants below the threshold of intake (<2/3 RNI) (Table 2), and the optimum accuracy levels (Table 3) imply that CIMI-Ethiopia could be a more reliable tool for dietary assessment in Southern Ethiopia. After improvement in the data entry mode and further validation studies in other regions, it could be an interesting solution for a faster and accurate assessment of individual nutrient intakes in Ethiopia.

### 4.2. Seasonal Variation in Nutrient Intake

The present study found a significant reduction in the mean intake of iron, zinc, magnesium, pantothenic acid, vitamin B1, protein, and energy in the lean wet season (Table 4). Likewise, limited prior research studies in Ethiopia have also reported a decline in energy intake in the lean season due to reduced food availability and increased food prices [32,33]. A very recent study reported a reduction in pulse/legume consumption in the lean wet season [28], which could be the first possible cause for the observed decrease in the nutrient intakes. The second could be a decline in animal source food consumption, together with a decrease in meal frequency in this season [28]. 

Literature indicates a risk of vitamin D deficiency relative to bone health in Ethiopia, which is a country with sunshine during all months of the year. For instance, a research study from Southern Ethiopia reported that only ~16% of women had 25-(OH)-vitamin D levels above 50 nmol/L [34]. Another study on school children from Central Ethiopia showed that 42% are vitamin D deficient [35]. For this deficiency problem, sufficient exposure to sun and the consumption of vitamin D-rich food are vital solutions. However, the current result shows little or no intake of vitamin D from the diet in a day preceding the survey in the postharvest dry season, and a further decline in lean wet season (Table 4). The observed high gap in vitamin D intake could be due to a lack of animal source foods and a limited consumption of eggs in both seasons [28]. Gebreegziabher and Stoecker also reported the absence of vitamin D-rich foods, fortified foods, and dietary supplements in the diets of women in a comparable agroecological zone [34]. 

Similar to vitamin D, the intake of vitamin B12 is very low across seasons, and almost absent in the lean wet season (Table 4). The possible reason for this could be a low intake of animal source foods (ASF) in the study population, and its further decline in the lean wet season [28]. This is because ASF are the usual sources of this vitamin [36]. Such a poor intake of foods rich in vitamin B12 could lead to its deficiency, which is a well-known cause of megaloblastic anemia that can occur at any age [37]. Therefore, it would be helpful to improve the consumption of animal source foods across seasons.

The increase in calcium and vitamin A intake in the lean wet season (Table 4) is associated with an increased intake of green leafy vegetables like Ethiopian kale [28], which is rich in them [38]. However, it was found that the percentage of participants below the threshold of an inadequate intake of calcium was over 85% in both seasons, which is supported by a previous report (74%) from a weighed food record study on pregnant women from the same agroecological zone [7]. Such a high prevalence of inadequate calcium intake from the diet may account for pre-eclampsia and eclampsia [39], and could adversely affect bone health [40]. Therefore, it is advisable to consume milk every day for pregnant women [41], and increase the consumption of teff and kale, which are rich in calcium [38].

This study has several strengths and few weaknesses. This is the first study of its kind in Southern Ethiopia. A large sample size and the comparison of the two seasons make it robust. However, the validation study was only conducted in rural communities. In addition, the accuracy of CIMI-Ethiopia was sometimes negatively affected by its data entry mode: 15 different portion sizes are available per food group, where differences between two portion sizes get higher the bigger the portion size is. Consequently, the intake of larger amounts of a food group is linked to a higher risk of over- or underestimation of the consumed foods in this group. Therefore, additional improvements in the data entry mode, for instance, changing the nonlinear stop point sliders to a linear one, could increase the accuracy of CIMI-Ethiopia further. Possible recall bias, and interviewer bias in 24HR [13] may over- or underestimate the nutrient intakes. The use of vitamin A fortified palm oil in this study may overestimate the vitamin A intake computed in a target population. However, such limitations may not have substantially affected our conclusions as we used the data sets to compare CIMI-Ethiopia vs. Nutrisurvey Programs, and assess variations in nutrient intakes in the postharvest dry season and lean wet season.

## 5. Conclusions

It has been found that CIMI-Ethiopia is valid and rapidly estimates the nutrient intake in Southern Ethiopia. The results show that the program could be a good solution to assess individuals with an inadequate nutrient intake in the region with a further improvement in accuracy. Additionally, the study demonstrates a significant reduction in the mean intakes of iron; zinc; magnesium; pantothenic acid; vitamin B1, B12, and D; protein; and energy in the lean wet season. For long term improvement of the maternal nutrient intake, regional food production should be enhanced by agricultural training, livestock breeding, and water conservation. Nutrition education should also be integrated into curricula for teachers. To achieve short-term improvements in the maternal nutrient intake, opportunities for food fortification and supplementation should be taken into account.

## Figures and Tables

**Table 1:** Sociodemographic characteristics of participants (N = 577).

Variable	Category	Number	Percent
Age of mothers, years	18–24	52	9.0
	25–34	512	88.7
	≥35	13	2.3
Lactating, postharvest dry season	Yes	433	75.0
	No	144	25.0
Lactating, lean wet season	Yes	338	58.6
	No	239	41.4
Marital status	Co-habiting in marriage	563	97.6
	Other	14	2.4
Ethnicity	Sidama	560	97.1
	Others	17	2.9
Religion	Protestants	528	91.5
	Others	49	8.5
Educational level	Illiterate	239	41.4
	Some education	338	58.6
Occupation	Housewives	506	87.7
	Others	71	12.3
Family size	3–5 members	349	60.5
	≥6 members	228	39.5

**Table 2:** Mean (SD), median (25th, 75th percentile), and number (percent) of participants below the threshold of an inadequate nutrient intake (<2/3 RNI) (N = 577).

Nutrient	Devi-ces	Postharvest Dry Season			Lean Wet Season		
		Mean (SD)	Median (25th, 75th Perc.)	N (%) of <2/3 RNI	Mean (SD)	Median (25th, 75th Perc.)	N (%) of <2/3 RNI
Iron (mg)	CIMI-E	30.1 (14.3)	27.1 (20.2, 36.6)	218 (37.8)	26.9 (11.4)	25.7 (19.8, 31.1)	291 (50.4)
	NS	32.9 (15.1)	29.5 (22.4, 39.7)	185 (32.1)	29.9 (11.6)	28.7 (21.9, 35.2)	252 (43.7)
Zinc (mg)	CIMI-E	7.1 (4.6)	6.2 (3.6, 9.6)	446 (77.3)	5.8 (3.8)	5.1 (3.0, 8.2)	465 (80.6)
	NS	6.5 (4.3)	5.6 (3.5, 8.4)	473 (82.0)	5.3 (3.4)	4.8 (2.8, 6.9)	480 (83.2)
Calcium (mg)	CIMI-E	464 (249)	432 (282, 606)	556 (96.4)	502 (233)	477 (342, 645)	560 (97.1)
	NS	547 (333)	481 (298, 733)	521 (90.3)	652 (340)	625 (400, 840)	489 (84.7)
Magnesium (mg)	CIMI-E	371 (216)	330 (215, 483)	132 (22.9)	329 (179)	293 (199, 438)	163 (28.2)
	NS	397 (227)	353 (232, 510)	115 (19.9)	340 (179)	314 (203, 451)	146 (25.3)
Niacin (mg)	CIMI-E	10.3 (4.7)	9.8 (6.9, 12.8)	338 (58.6)	10.1 (4.6)	9.5 (6.6, 13.0)	337 (58.4)
	NS	12.4 (5.1)	11.9 (8.8,15.3)	236 (40.9)	12.1 (4.7)	11.3 (8.5, 15.1)	248 (43.0)
Vitamin A (μg)	CIMI-E	789 (772)	608 (141, 1361)	274 (47.5)	1092 (842)	930 (363, 1574)	160 (27.7)
	NS	1108 (1136)	680 (218, 1951)	255 (44.2)	1509 (1143)	1338 (513, 2174)	137 (23.7)
PA (mg)	CIMI-E	2.4 (1.5)	2.2 (1.3, 3.3)	505 (87.5)	2.0 (1.3)	1.9 (1.1, 2.8)	527 (91.3)
	NS	2.5 (1.7)	2.1 (1.3, 3.3)	494 (85.6)	2.0 (1.3)	1.8 (1.1, 2.6)	531 (92.0)
Vitamin B12 (μg)	CIMI-E	0.019 (0.158)	0.000 (0.000, 0.000)	575 (99.7)	0.001 (0.012)	0.000 (0.000, 0.000)	577 (100)
	NS	0.021 (0.194)	0.000 (0.000, 0.000)	574 (99.5)	0.001 (0.012)	0.000 (0.000, 0.000)	577 (100)
Vitamin B1 (mg)	CIMI-E	1.0 (0.5)	0.9 (0.6, 1.3)	270 (46.8)	0.9 (0.5)	0.8 (0.5, 1.2)	297 (51.5)
	NS	1.0 (0.5)	0.9 (0.7, 1.3)	249 (43.2)	0.9 (0.4)	0.9 (0.6, 1.2)	274 (47.5)
Vitamin B6 (mg)	CIMI-E	1.2 (0.7)	1.1 (0.7, 1.6)	310 (53.7)	1.1 (0.6)	1.0 (0.7, 1.5)	325 (56.3)
	NS	1.4 (0.9)	1.2 (0.8, 1.8)	277 (48.0)	1.3 (0.7)	1.2 (0.8, 1.7)	271 (47.0)
Vitamin D (μg)	CIMI-E	0.015 (0.011)	0.014 (0.006, 0.020)	577 (100)	0.015 (0.014)	0.014 (0.000, 0.023)	577 (100)
	NS	0.003 (0.013)	0.000 (0.000, 0.000)	577 (100)	0.002 (0.012)	0.000 (0.000, 0.000)	577 (100)
Protein (g)	CIMI-E	35.9 (20.6)	33.1 (19.7, 46.6)	299 (51.8)	31.1 (18.2)	28.0 (16.8, 42.7)	339 (58.8)
	NS	36.8 (20.0)	33.7 (21.9, 47.0)	290 (50.3)	32.7 (17.8)	30.0 (19.2, 43.3)	312 (54.1)
Energy (kcal)	CIMI-E	1531 (588)	1454 (1100, 1881)	380 (65.9)	1405 (507)	1335 (1068, 1706)	402 (69.7)
	NS	1596 (587)	1530 (1148, 1946)	354 (61.4)	1495 (508)	1422 (1149, 1819)	370 (64.1)

Perc.: percentile; PA: pantothenic acid; SD: standard deviation; CIMI-E: calculator of inadequate micronutrient intake program for Ethiopia; NS: NutriSurvey; seasonal difference in micronutrient intake is significant at *p*-value of 0.05.

**Table 3:** Average mean and standard deviation (SD) of the difference between the nutrient intake computed by CIMI-Ethiopia minus the intake calculated by NS in two seasons, and the Pearson’s coefficients (r) between intakes computed by CIMI-Ethiopia and NS (N = 1154).

Nutrient	Mean Difference	SD	% Difference *	r
Iron (mg)	−2.87	4.90	9.23 ^b^	0.93 **
Zinc (mg)	0.56	1.36	9.45 ^b^	0.95 **
Calcium (mg)	−116.53	161.87	19.43 ^c^	0.90 **
Magnesium (mg)	−18.75	57.77	5.09 ^b^	0.96 **
Niacin (mg)	−2.05	1.43	16.75 ^c^	0.96 **
Vitamin A (μg)	−367.65	526.25	28.10 ^c^	0.91 **
Pantothenic acid (mg)	−0.005	0.58	0.22 ^a^	0.92 **
Vitamin B12 (μg)	−0.001	0.048	10.00 ^b^	0.95 **
Vitamin B1 (mg)	−0.02	0.15	2.06 ^a^	0.95 **
Vitamin B6 (mg)	0.17	0.27	12.59 ^b^	0.95 **
Vitamin D (μg)	0.012	0.015	NA	0.21 **
Protein (g)	−1.26	5.05	3.62 ^a^	0.97 **
Energy (kcal)	−77.23	147.97	5.00 ^b^	0.96 **

* = difference expressed as percent of NS (NutriSurvey) result; NS: NutriSurvey; ^a^ = very high accuracy; ^b^ = good accuracy; ^c^ = moderate accuracy; NA: data not appropriate for analysis; ** Correlation between CIMI-Ethiopia and NS is significant at *p*-value < 0.001; CIMI-Ethiopia: Calculator of inadequate micronutrient intake program for Ethiopia.

**Table 4:** Seasonal variation in nutrient intakes in postharvest dry and lean wet seasons (N = 577) computed by NutriSurvey 2007.

Nutrient	Postharvest Dry Season		Lean Wet Season		*p*-Value
	Mean (SD)	Median (25th, 75th perc.)	Mean (SD)	Median (25th, 75th perc.)	
Iron (mg)	32.9 (15.1)	29.5 (22.4, 39.7)	29.9 (11.6)	28.7 (21.9, 35.2)	<0.001
Zinc (mg)	6.5 (4.3)	5.6 (3.5, 8.4)	5.3 (3.4)	4.8 (2.8, 6.9)	<0.001
Calcium (mg)	547 (333)	481 (298, 733)	652 (340)	625 (400, 840)	<0.001
Magnesium (mg)	397 (227)	353 (232, 510)	340 (179)	314 (203, 451)	<0.001
Niacin (mg)	12.4 (5.1)	11.9 (8.8,15.3)	12.1 (4.7)	11.3 (8.5, 15.1)	0.394
Vitamin A (μg)	1108 (1136)	680 (218, 1951)	1509 (1143)	1338 (513, 2174)	<0.001
Pantothenic acid (mg)	2.5 (1.7)	2.1 (1.3, 3.3)	2.0 (1.3)	1.8 (1.1, 2.6)	<0.001
Vitamin B12 (μg)	0.021 (0.194)	0.000 (0.000, 0.000)	0.001 (0.012)	0.000 (0.000, 0.000)	0.007
Vitamin B1 (mg)	1.0 (0.5)	0.9 (0.7, 1.3)	0.9 (0.4)	0.9 (0.6, 1.2)	0.001
Vitamin B6 (mg)	1.4 (0.9)	1.2 (0.8, 1.8)	1.3 (0.7)	1.2 (0.8, 1.7)	0.076
Vitamin D (μg)	0.003 (0.013)	0.000 (0.000, 0.000)	0.002 (0.012)	0.000 (0.000, 0.000)	0.009
Protein (g)	36.8 (20.0)	33.7 (21.9, 47.0)	32.7 (17.8)	30.0 (19.2, 43.3)	<0.001
Energy (kcal)	1596 (587)	1530 (1148, 1946)	1495 (508)	1422 (1149, 1819)	0.020

Wilcoxon signed-rank test was used for data analysis; SD: standard deviation; per.: percentile; seasonal difference in nutrient intake is significant at *p*-value of 0.05.

## References

[1] Tulchinsky T.H. (2010). Micronutrient Deficiency Conditions: Global Health Issues. BMC Public Health Rev..

[2] WHO, WFP, UNICEF (2007). Preventing and Controlling Micronutrient Deficiencies in Populations Affected by an Emergency: Multiple Vitamin and Mineral Supplements for Pregnant and Lactating Women, and for Children Aged 6 to 59 Months.

[3] Gani G., Beenish G., Bashir O., Bhat T.A., Naseer B., Qadri T., Jan N. (2018). Hidden hunger and its prevention by food processing: A review. IJUIM.

[4] Brandt S.A., Spring A., Hiebsch C., Yntiso G., Tabogie E., Diro M., Wolde-Michael G., Tesfaye S., McCabe J.T., Shigeta M. (1997). The “Tree Against Hunger” Enset-Based Agricultural Systems in Ethiopia.

[5] Gebremedhin S., Enquselassie F., Umeta M. (2011). Prevalence of prenatal zinc deficiency and its association with socio-demographic, dietary and health care related factors in Rural Sidama, Southern Ethiopia: A cross-sectional study. BMC Public Health.

[6] Gebreselassie S.G., Gase F.E., Deressa M.U. (2013). Prevalence and Correlates of Prenatal Vitamin A Deficiency in Rural Sidama, Southern Ethiopia. J. Health Popul. Nutr..

[7] Abebe Y., Bogale A., Hambidge K.M., Stoecker B.J., Arbide I., Teshome A., Krebs N.F., Westcott J.E., Bailey K.B. (2008). Inadequate intakes of dietary zinc among pregnant women from subsistence households in Sidama, Southern Ethiopia. Public Health Nutr..

[8] Borrell J.S., Biswas M.K., Goodwin M., Blomme G., Schwarzacher T., Heslop-Harrison J.S., Wendawek A.M., Berhanu A., Kallow S., Janssens S. (2019). Enset in Ethiopia: A poorly characterized but resilient starch staple. Ann. Bot..

[9] Daba T., Shigeta M. (2016). Enset (Ensete Ventricosum) Production in Ethiopia: Its Nutritional and Socio-Cultural Values. Agric. Food Sci. Res..

[10] Karssa T., Papini A. (2018). Effect of Clonal Variation on Quality of Kocho, Traditional Fermented Food from Enset (Ensete Ventricosum), Musaceae. Int. J. Food Sci. Nutr. Eng..

[11] Bezuneh T., Feleke A. (1966). The production and utilization of the genus ensete in Ethiopia. Econ. Bot..

[12] Abebe Y., Stoecker B.J., Hinds M.J., Gates G.E. (2006). Nutritive value and sensory acceptability of corn- and kocho- based foods supplemented with legumes for infant feeding in Southern Ethiopia. Afr. J. Food Agric. Nutr. Dev..

[13] Shim J., Oh K., Kim H.C. (2014). Dietary assessment methods in epidemiologic studies. Epidemiol. Health.

[14] Jati I.R.A.P., Widmer C., Purwestri R.C., Wirawan N.N., Gola U., Lambert C., Biesalski H.K. (2014). Design and validation of a program to identify inadequate intake of iron, zinc, and vitamin A. Nutrition.

[15] Wald J.P., Asare E., Nakua E.K., Lambert C., Biesalski H.K., Gola U., Nohr D. Dietary assessment using the CIMI approach: A case study from three districts of the Ashanti region in Ghana. Proceedings of the 3rd International Conference on Global Food Security.

[16] Wald J.P., Asare E., Nakua E.K., Nohr D., Lambert C., Riedel S., Gola U., Biesalski H.K. (2019). Validation of a computer-based analysis tool for real-time dietary assessment within a Ghanaian region. NFS J..

[17] Lambert C., Eleraky L., Mbwana H., Kinabo J., Biesalski H.K., Riedel S., Widmer C., Gola U., Stuetz W. Validation of a food group based nutrition software to assess nutrient intake in Tanzania. Proceedings of the Tropentag Conference.

[18] Desalegn B., Borko T., Lambert C., Widmer C., Gola U., Riedel S., Negese T., Biesalski H.K. (2019). Nutrient Intake of 12–59-Months-Old Children and Women in Ethiopia, and Development of Calculator for Inadequate Micronutrient Intake (CIMI) App for Ethiopian Population. Curr. Dev. Nutr..

[19] Desalegn B.B., Lambert C., Riedel S., Negese T., Biesalski H.K. (2018). Ethiopian Orthodox Fasting and Lactating Mothers: Longitudinal Study on Dietary Pattern and Nutritional Status in Rural Tigray, Ethiopia. Int. J. Environ. Res. Public Health.

[20] Ethiopian Public Health Institute [EPHI] (2013). Ethiopia National Food Consumption Survey. https://www.ephi.gov.et/images/pictures/National%20Food%20Consumption%20Survey%20.

[21] Erhard J. (2007). NutriSurvey for Windows. https://www.nutrisurvey.de.

[22] World Health Organization (WHO), Food and Agriculture Organization (FAO) (2004). Vitamin and Mineral Requirements in Human Nutrition.

[23] WHO (2007). Protein and Amino Acid Requirements in Human Nutrition.

[24] FAO, WHO, UNU (2004). Human Energy Requirements. https://www.who.int/nutrition/publications/nutrientrequirements/9251052123/en/.

[25] CSA Population Projection of Ethiopia for All Regions at Wereda Level from 2014–2017.

[26] Quinlan R.J., Quinlan M.B., Dira S., Caudell M., Sooge A., Assoma A.A. (2015). Vulnerability and Resilience of Sidama Enset and Maize Farms in Southwestern Ethiopia. J. Ethnobiol..

[27] Zewdie E., Sivakumar S. (2017). Determinants of Off Farm Participation of Rural Farm Households in Shebedino District of Sidama. Int. J. Dev. Res..

[28] Bosha T., Lambert C., Riedel S., Melesse A., Biesalski H.K. (2019). Dietary Diversity and Anthropometric Status of Mother–Child Pairs from Enset (False Banana) Staple Areas: A Panel Evidence from Southern Ethiopia. Int. J. Environ. Res. Public Health.

[29] Hulley S.B., Cummings S.R., Browner W.S., Grady D.G., Newman T.B., Williams L., Wilkins (2013). Designing Clinical Research. A Wolters Kluwer Business.

[30] Liabsuetrakul T., Kuning M., Sukchan P., Chongsuvivatwong V., Sornsrivichai V., Songwathana P. (2010). Inadequacy of nutrients intake among pregnant women in the Deep South of Thailand. BMC Public Health.

[31] Serra-Majem L., Ribas L., Garcia A., Perez-Rodrigo C., Aranceta J. (2003). Nutrient adequacy and Mediterranean Diet in Spanish school children and adolescents. Eur. J. Clin. Nutr..

[32] Hirvonen K., Taffesse A.S., Hassen I.W. (2016). Seasonality and household diets in Ethiopia. Public Health Nutr..

[33] Sibhatu K.T., Qaim M. (2017). Rural food security, subsistence agriculture, and seasonality. PLoS ONE.

[34] Gebreegziabher T., Stoecker B.J. (2013). Vitamin D insufficiency in a sunshine-sufficient area: Southern Ethiopia. Food Nutr. Bull..

[35] Wakayo T., Belachew T., Vatanparast H., Whiting S.J. (2015). Vitamin D Deficiency and Its Predictorsina Country with Thirteen Months of Sunshine: The Case of School Children in Central Ethiopia. PLoS ONE.

[36] Watanabe F., Yabuta Y., Bito T., Teng F. (2014). Vitamin B12-containing plant food sources for vegetarians. Nutrients.

[37] Green R. (2019). Vitamin B12 deficiency from the perspective of a practicing hematologist. Am. Soc. Hematol..

[38] Umeta M., West C.E., Fufa H. (2005). Content of zinc, iron, calcium and their absorption inhibitors in foods commonly consumed in Ethiopia. J. Food Compost. Anal..

[39] Hofmeyr G.J., Betrán A.P., Singata-madliki M., Cormick G., Munjanja S.P., Fawcus S., Mose S., Hall D., Ciganda A., Seuc A.H. (2019). Prepregnancy and early pregnancy calcium supplementation among women at high risk of pre-eclampsia: A multicentre, double-blind, randomised, placebo-controlled trial. Lancet.

[40] Balk E.M., Adam G.P., Langberg V.N., Earley A., Clark P., Ebeling P.R. (2017). Global dietary calcium intake among adults: A systematic review. Osteoporos. Int..

[41] Chotboon C., Soontrapa S., Buppasiri P., Muktabhant B., Kongwattanakul K., Thinkhamrop J. (2018). Adequacy of calcium intake during pregnancy in a tertiary care center. Int. J. Women’s Health.

